# Nutrient Intake among Pregnant Women in Spain and Poland: A Comparative Analysis

**DOI:** 10.3390/nu15143225

**Published:** 2023-07-20

**Authors:** Lucía Iglesias-Vázquez, Joanna Suliburska, Rafał Kocyłowski, Ewa Bakinowska, Victoria Arija

**Affiliations:** 1 Nutrition and Mental Health (NUTRISAM) Research Group, Universitat Rovira i Virgili, 43204 Reus, Spain; lucia.iglesias@urv.cat; 2 Institut d’Investigació Sanitaria Pere Virgili (IISPV), 43204 Reus, Spain; 3 Department of Human Nutrition and Dietetics, Poznan University of Life Sciences, 60-637 Poznan, Poland; joanna.suliburska@up.poznan.pl; 4 PreMediCare New Med Medical Center, 61-693 Poznan, Poland; rkocylow@gmail.com; 5 Institute of Mathematics, Poznan University of Technology, 60-965 Poznan, Poland; ewa.bakinowska@put.poznan.pl; 6 Collaborative Research Group on Lifestyles, Nutrition, and Smoking (CENIT), Tarragona-Reus Research Support Unit, IDIAP Jordi Gol, 43003 Tarragona, Spain

**Keywords:** diet, pregnancy, dietary guidelines, nutritional intake, recommended dietary allowance

## Abstract

Prenatal nutrition plays a crucial role in maternal and child health. This study aims to compare nutrient intake and its adequacy to recommendations among pregnant women in Spain and Poland. The ECLIPSES study in Spain utilized a self-administered food frequency questionnaire, while the PREDISH study in Poland employed a 3-day interview method. We assessed energy and nutrient intake against recommended dietary allowances. The analysis included 583 participants in the first trimester and 465 participants in the third trimester from both countries. Our findings revealed insufficient intake of iron, vitamin D, and vitamin B9 among pregnant women in both Spain and Poland. Significant differences were observed in the intake of energy, carbohydrates, fiber, calcium, iron, and vitamins D, E, C, B6, B9, and B12. Notably, 81.6% and 21.5% of participants did not meet the recommended minimum carbohydrate intake, while 99.8% and 43.8% exceeded the limit for total fat, particularly monounsaturated fatty acids (MUFAs). Tailored dietary guidance based on regional differences is crucial for pregnant women. Although variations in dietary intake were observed, both Spain and Poland faced similar risks of nutritional deficiencies, particularly for iron, vitamin D, and vitamin B9. These findings emphasize the need for enhanced efforts in preventing these deficiencies and promoting optimal prenatal nutrition.

## 1. Introduction

Prenatal nutrition plays a crucial role in the proper development of pregnancy, maternal health, and the growth and development of the fetus [1]. Taking care of maternal dietary habits and optimal nutrient intake is of great importance to prevent nutritional imbalances, which can be detrimental to maternal and infant health and even lead to mid- and long-term consequences, such as low birth weight, fetal growth restriction and risk of chronic diseases later in life [1,2,3,4,5,6,7,8,9]. It is, therefore, common to find guidelines recommending healthy eating behaviors during pregnancy to optimize maternal and fetal health [10,11,12]. Recommendations are generally similar, especially among developed countries, while dietary patterns differ widely depending on economics, culture, or food availability. Thus, recommendations for food consumption and nutrient intake may be achieved to a lesser or greater extent depending on the composition of the diet in each country.

The Mediterranean diet, for instance, which is the most common dietary pattern in Spain, is well-known for its abundance of fruits, vegetables, whole grains, legumes, nuts, and olive oil, and a moderate intake of fish, dairy products, and red wine [13,14]. It is typically low in saturated fat, cholesterol, and sodium, while rich in antioxidants, fiber, and omega-3 fatty acids and has been associated with various health benefits, including a reduced risk of cardiovascular disease, type 2 diabetes, and some cancers [13,15]. Another example is the Polish diet, which is traditionally based on cereals, potatoes, vegetables, and meat. Although it is high in some nutrients that have been linked to certain health risks, such as saturated fat and sodium, the Polish diet also offers several benefits when consumed in moderation as part of a balanced diet, including being nutrient-dense, and high in protein and fiber [16,17].

Considering that different types of diets can lead to different nutritional states, including different risks of nutritional deficiencies, dietary advice should be adapted to the actual situation of each population, based on the extent to which pregnant women reach the recommended intake in each country. However, dietary intake assessment is not common during pregnancy and many nutritional deficiencies undergo undiagnosed, leading to health problems in mother and child. This paper aimed to describe the dietary intake and its adequacy to the recommendations of pregnant women from Spain and Poland, two geographically distant European countries with different lifestyles and cultural backgrounds. This knowledge will make it possible to identify areas for improvement and provide advice for optimizing maternal and fetal health.

## 2. Materials and Methods

This work included data from women participating in the ECLIPSES study (Tarragona, Spain, 2013–2017) and the PREDISH study (Poznan, Poland, 2016–2018). The ECLIPSES study is a longitudinal study that assesses the dietary intake of healthy pregnant women recruited in early pregnancy and followed up throughout the whole gestation. Exclusion criteria were as follows: multiple pregnancies, adverse obstetric history, use of >10 mg iron daily during the three months before week 12 of gestation and previous severe illness (immunosuppression) or chronic disease that could affect the nutritional status (cancer, diabetes, malabsorption, and liver disease). Extended information can be found elsewhere [18,19]. The PREDISH study is an observational study mainly aimed at prospectively assessing the dietary intake of participating pregnant women, with particular emphasis on the supply of minerals. Exclusion criteria included genetic defects of the pregnant woman and/or fetus, multiple pregnancies, use of drugs affecting the mineral balance in the body, and maternal exposure to alcohol, drugs, or tobacco smoke. The PREDISH study considered a pool of participants in the first trimester of pregnancy and another pool of different participants in the third trimester. The studies were registered in ClinicalTrials.gov: ECLIPSES study (NCT03196882) and PREDISH study (NCT03598361).

The ECLIPSES study assessed eating habits through a self-administered food frequency questionnaire (FFQ) (Supplementary File S1) previously validated in the Spanish population [20]. Participants reported usual food consumption retrospectively at weeks 12 and 36 of pregnancy. The FFQ was explained by specialized midwives and, subsequently, revised and analyzed by nutritionists. From the information obtained by FFQ, the daily consumption frequency was calculated. Then, the size and weight of a serving portion were standardized according to the validation questionnaire [20] and each food item was calculated in grams per day. From this information, energy (kcal/day), macronutrients (g/day) and micronutrients (mg/day or µg/day) intake was calculated by using the REGAL (Répertoire Général des Aliments) food consumption table [21] and the Mataix Verdú Spanish food composition table [22], both adapted to the questionnaire items. In the PREDISH study, the dietary assessment was measured using a 3-day nutritional interview. The results were analyzed using Dietetyk 2015.1 software.

The energy and nutrient intake were compared with the recommended dietary allowance (RDA) for energy and each nutrient as outlined in the dietary reference intakes recommended by the US Institute of Medicine (IOM) [23]. Adequacy to the RDAs was defined as follows: excessive intake (>120%), adequate intake (>80–120%), close to adequate intake (40–80%), and inadequate intake (<40%). Additionally, the energy structure from nutrients was calculated and compared to the recommendations from the IOM [18], that is, protein 10%–35%, carbohydrates 45–65%, total fats 20–35%, saturated fatty acids (SFAs) < 10%, and polyunsaturated fatty acids (PUFAs) 5–10%. As for MUFAs intake, only a few organizations give quantitative recommendations, ranging from 10–25% of total energy intake according to a scoping review of current guidelines [24].

In both studies, other variables were recorded, including age, anthropometric measurements, and educational level. Height was measured by research staff while pre-pregnancy weight was self-reported; from this data, pre-pregnancy body mass index (BMI) was calculated. As for the family socioeconomic status (SES), in the ECLIPSES study it was calculated using the participants’ and partners’ educational level and occupational status [18] while in the PREDISH study, a questionnaire devised by the study researchers and validated was used.

The results were expressed as the mean and standard deviation (SD) for quantitative variables and as percentages for qualitative variables. To compare means of energy and nutrient intake, *t*-tests between the groups were used while χ^2^ tests were used for comparing the proportion of participants having an adequate intake between the groups. The statistical analyses were done using SPSS version 25 and statistical significance was determined by a *p*-value of <0.05.

The ECLIPSES study was designed in agreement with the Declaration of Helsinki/Tokyo and was approved by the Clinical Research Ethics Committee of the Jordi Gol University Institute for Primary Care Research and the Pere Virgili Health Research Institute (approval ID: 118/2017). The ECLIPSES study was registered at www.clinicaltrialsregister.eu as EudraCT number 2012-005480-28 and at www.clinicaltrials.gov as NCT03196882. The research on the PREDISH study was carried out with the approval of the Local Bioethics Commission at the Poznań University of Medical Sciences (approval no 30/15).

## 3. Results

**Figure 1:**
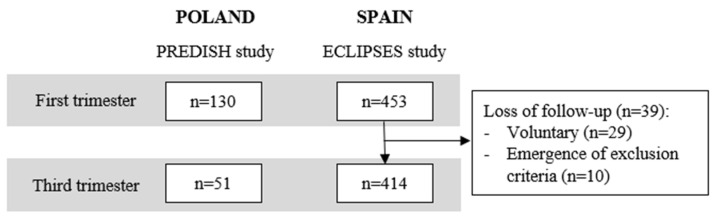
Flowchart of the studies.

A total of 583 pregnant women were included in the study in the first trimester (453 from Spain and 130 from Poland) and 465 women were included in the third trimester (414 from Spain and 51 from Poland) (Figure 1). The baseline characteristics of the participating women are presented in Table 1. The median age of women in both countries was 31 years during the first trimester. Spanish participants had a significantly higher mean BMI compared to Polish ones during the first trimester (24.74 kg/m^2^ vs. 22.99 kg/m^2^, *p* < 0.001), but not during the third trimester (24.71 kg/m^2^ vs. 23.33 kg/m^2^, *p* = 0.078). Significant differences were found in the educational level. Regarding socioeconomic status, statistically significant differences were only found between Spanish and Polish participants during the first trimester.

Table 2 provides information on the dietary intake of pregnant women from Spain and Poland during the first and third trimesters of gestation. Spanish pregnant women consumed significantly more energy, carbohydrates, and fats in the first trimester than Polish pregnant women. However, Polish pregnant women consumed significantly more protein, fiber, and vitamins B6 and B9 than Spanish pregnant women. In the third trimester, the two countries had no significant differences in energy intake. However, Spanish pregnant women consumed significantly more carbohydrates and monounsaturated fats, while Polish pregnant women consumed significantly more proteins, free sugars, fiber, iron, vitamin B6, and vitamin B9 than Spanish pregnant women.

When the adequacy was assessed based on the Institute of Medicine’s recommendations (Table 3), adequate intake (>80–120% of RDA) during the first trimester of pregnancy was found for proteins (82.04%), carbohydrates (102.55%), vitamin C (94.66%), and vitamin B2 (96.90%) among Spanish participants. For Polish participants, it was found for proteins (84.68%), carbohydrates (114.74%), and vitamins B2 (90.37%), B6 (93.48%), and B12 (108.57%). Excessive intake (>120% of RDA) was found among both Spanish women for vitamin B12 (170.23%). However, intakes were far from meeting RDAs (below 40%) among study populations from both Spain and Poland for iron (29.04% and 31.58%), vitamin D (12.18% and 17.11%), and vitamin B9 (35.72% and 30.33%). Statistically significant differences were found between countries for energy, carbohydrates, fiber, calcium, iron, and vitamins D, E, C, B6, B9, and B12. During the third trimester of pregnancy, adequate intake was found for carbohydrates (94.54%), vitamin C (87.06%), and vitamin B2 (95.82%) among Spanish participants; and for proteins (101.82%), and vitamins C (95.10%), B2 (105.54%), and B6 (111.71%) among Polish women. Excessive intake was found for vitamin B12 (170.60%) among Spanish women and for carbohydrates (140.95%) and vitamin B12 (152.84%) among Polish participants. However, the intakes were below 40% of RDA both in Spain and Poland for iron (27.34% and 36.94%), vitamin D (11.92% and 27.63%), and vitamin B9 (33.07% and 35.22%). Statistically significant differences were found between countries for proteins, carbohydrates, fiber, iron, and vitamins D, E, B1, B2, and B6.

**Figure 2:**
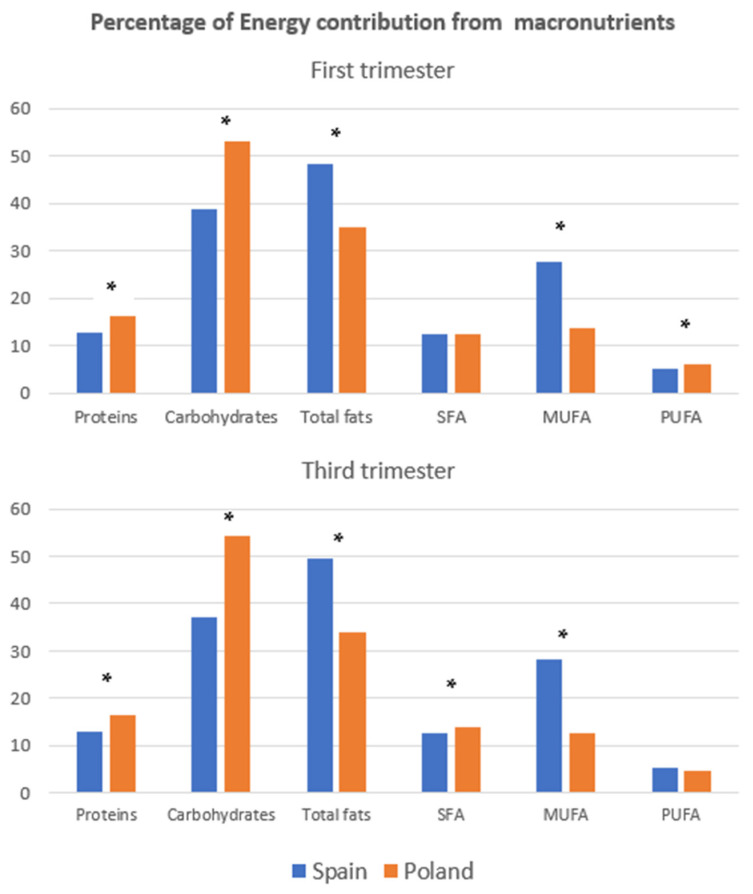
Energy structure from macronutrients in Spanish and Polish women. SFA, saturated fatty acids; MUFA, monounsaturated fatty acids; PUFA, polyunsaturated fatty acids. * Statistically significant difference.

Regarding the average percentage of energy contributed by macronutrients in pregnancy in Spanish and Polish participants, respectively, in the first trimester, 12.8% and 16.27% were from proteins, 38.63% and 52.94% were from carbohydrates, and 48.32% and 35.08% were from total fats, with 99.8% of Spanish participantsabove the recommended range. Moreover, regarding fatty acids, saturated fatty acids (SFAs) represented 12.28% and 12.53% of total energy, monounsaturated fatty acids (MUFAs) represented 27.54% and 13.61%, and polyunsaturated fatty acids (PUFAs) represented 5.14% and 6.11% in study populations from Spain and Poland, respectively (Figure 2). When compared to the recommended guidelines, 81.6% and 21.5% of Spanish and Polish participantsdid not reach the lower threshold of carbohydrate intake. For total fat intake, 99.8% and 43.8% of participants in each country exceeded the upper limit of total fats, with the highest difference observed in the intake of MUFAs, with Spanish participantsexceeding the recommended limit by 71%, while Polish participantsexceeded it by 3.8%. Very similar results were found for the third trimester (Table 4).

## 4. Discussion

The present study provides valuable insights into the differences in nutrition during pregnancy between women of different European countries: Spain, located in southern Europe, specifically in the Mediterranean basin; and Poland, located in Eastern Europe. Based on the assumption that dietary patterns are different, the adequacy to the recommended nutritional intake is in most cases adequate or close to adequacy, although with specific differences. Spanish pregnant participantsconsumed more energy from fat, but mainly from a healthy type of fat, such as MUFAs, and ingested little fiber. On the other hand, Polish participantsconsumed a higher amount of carbohydrates, specifically in the third trimester of pregnancy. Participating women in both countries were at high risk of inadequate intake of iron, vitamin D, and vitamin B9.

In comparison to the recommended guidelines [23], a significant proportion of Spanish and Polish participantshave inadequate carbohydrate intake, with 81.6% and 21.5% respectively falling below the lower threshold. Additionally, a vast majority of women from both groups consume excessive amounts of total fat, with 99.8% and 43.8% exceeding the upper limit. The most notable disparity was observed in the intake of MUFAs, with Spanish women exceeding the recommended limit by 71%, while Polish participants exceeded it by 3.8%. Similar adequacy to the recommendations was observed in the first and third trimesters of pregnancy.

The higher intake of MUFAs observed among Spanish participantsboth in early and late pregnancy is likely due to adherence to the Mediterranean diet, high in olive oil and nuts which are the main source of this healthy fatty acid [15,25,26]. Their high vitamin intake also reflected their adherence to the Mediterranean diet, due to the richness of fruit and vegetables typical of this dietary pattern [13,27]. However, the high intake of free sugars and the high percentage of energy from fat in the diet of Spanish participantssuggests that the current Spanish diet is shifting away from the recommended Mediterranean pattern, according to the recent trend reported in other Mediterranean countries [28,29]. In this regard, it has been observed that fruit, vegetables, and cereals are consumed less and less, while the consumption of meat, dairy and sugary products has increased in recent times [28,30]. It poses a concern because it may result in excessive weight gain and an increased risk of several pregnancy complications, such as gestational diabetes and hypertension [31,32]. On the other hand, the traditional Polish diet tends to be rich in meat, which could explain the higher protein intake among participating Polish pregnant women compared to Spanish participants [33]. In addition, potatoes, cabbage, and cereals are also very common foods in the Polish diet, which explains the higher intake of carbohydrates and fiber among participantsin Poland than among those in Spain in the present study [16,17]. These findings are consistent with other studies that have reported high protein and fiber intake in Eastern Europe [34]. These features can result in benefits for mother-child health since adequate protein intake is essential for the growth and development of the fetus [35,36], while fiber intake can reduce cholesterol levels and the risk of insulin resistance [37]. Similar but slightly higher values of supply for macronutrients in Polish pregnant women were obtained in our previous study [38].

Beyond the differences in macronutrient intake, the nutritional risk observed in samples from both countries was the same. Insufficient intake (<40% of RDA) of iron, vitamin D and vitamin B9 were consistently detected among Spanish and Polish participants during pregnancy. Given that these nutrients are essential for fetal development, this is an issue of concern. Briefly, it is known that iron deficiency can lead to anemia, which can increase the risk of premature birth, low birth weight and other complications [39,40]; prenatal vitamin D deficiency has been linked to an increased risk of gestational diabetes, pre-eclampsia and premature birth [3,4,9], and can also affect calcium metabolism, leading to problems for bone health in both mother and child [41]; and finally, vitamin B9 insufficiency during pregnancy may results in preterm delivery, an increased risk of neural tube defects, and also impaired cognitive development in children [42,43,44]. However, inadequate intake and nutritional deficit of iron, vitamin D and vitamin B9 during pregnancy is not an isolated problem, but is widespread throughout Europe, as reported by current reviews [45,46,47]. In our previous study, we also observed the intake of iron, calcium, vitamin D, and folate below the recommended level in pregnant women [38]. Moreover, in the Polish population of pregnant women, the use of vitamin and mineral supplements increased their supply, but the supplementation was not sufficient to achieve the recommended levels of micronutrient intake.

This finding highlights the need for nutrition education programs to raise awareness of the importance of dietary habits during pregnancy to help ensure that women meet their nutritional needs. Future studies should make efforts in analyzing the dietary intake of pregnant women considering larger sample sizes in different countries, mainly focusing on iron, vitamin D and vitamin B9 deficiencies. Additionally, expert nutritional counselling and follow-up should be implemented during pregnancy at the primary care level.

This study has several strengths and limitations. The main point of the study was the extensive collection of data on dietary intake using validated and reliable questionnaires, which allowed for a comprehensive and comparable calculation of nutrient intakes between the two countries. However, dietary assessment through questionnaires is susceptible to misreporting bias, especially for unhealthy foods. Also, cooking methods and temperature play an important role in nutrient content and, unfortunately, participants did not receive specific instructions in this regard. Moreover, as the participants in Poland were not the same in the first and third trimesters of pregnancy, the data could only be analyzed cross-sectionally. This prevented us from describing the evolution of nutrient intake during pregnancy in both countries, which could have provided valuable information on women’s awareness of the importance of nutrition throughout pregnancy.

## 5. Conclusions

Based on the findings of this research study, it is evident that there are notable differences in nutritional intake during pregnancy between women from Spain and Poland, particularly in terms of carbohydrates, fats, and certain micronutrients. However, despite these differences in dietary patterns, it is important to recognize that both Spanish and Polish pregnant women encounter similar challenges when it comes to achieving recommended nutrient intakes during pregnancy. The inadequate intake of essential nutrients such as iron, vitamin D, and vitamin B9 is a common reality in both countries, which raises significant concerns regarding the potential adverse pregnancy outcomes associated with these nutritional deficiencies. The fact that these deficiencies persist in both regions highlights the urgency to address this issue and underscores the importance of targeted dietary advice for pregnant women. By tailoring dietary recommendations to the specific needs and preferences of pregnant women in different regions, we can enhance the relevance and effectiveness of nutritional interventions aimed at improving maternal and child health. This research underscores the significance of providing region-specific guidance, considering the cultural, dietary, and socioeconomic factors that contribute to divergent nutritional patterns. Overall, this study provides valuable insights into the nutritional disparities among pregnant women from Spain and Poland and emphasizes the critical need for tailored dietary advice to address the challenges faced by these populations. By addressing inadequate intakes of vital nutrients, particularly iron, vitamin D, and vitamin B9, we can potentially mitigate the adverse pregnancy outcomes associated with these deficiencies, ultimately promoting better maternal and child health outcomes in both countries.

## Figures and Tables

**Table 1:** Baseline characteristics of participating women.

	1st Trimester			3rd Trimester		
Characteristics	Spain (n = 453)	Poland (n = 130)	*p*	Spain (n = 414)	Poland (n = 51)	*p*
Age, years	31 [17–46]	31 [19–44]	0.269	31 [17–46]	32 [20–45]	0.198
Body mass index (kg/m^2^)	24.74 (4.61)	22.99 (3.60)	**<0.001**	24.71 (4.66)	23.33 (3.36)	0.078
Educational level						
Primary	31.40 [142]	13.85 [18]	**<0.001**	32.37 [134]	9.62 [5]	**0.001**
Secondary	40.90 [185]	43.08 [56]	0.648	41.06 [170]	36.54 [19]	0.601
Higher education	27.70 [126]	43.08 [56]	**0.001**	26.57 [110]	53.85 [27]	**<0.001**
Socioeconomic status						
Low	10.7 [48]	16.92 [22]	0.050	8.70 [36]	5.77 [3]	0.494
Middle	66.5 [301]	71.54 [93]	0.274	68.36 [283]	71.15 [36]	0.746
High	22.9 [104]	11.54 [15]	**0.004**	22.95 [95]	23.08 [12]	0.926

The data are shown in median [min–max], mean (standard deviation), and % [n]. Statistically significant differences are highlighted in bold.

**Table 2:** Dietary intake of pregnant women from Spain and Poland.

	1st Trimester			3rd Trimester		
	Spain (n = 452)	Poland (n = 130)	*p*	Spain (n = 414)	Poland (n = 51)	*p*
Energy (Kcal/d)	1806.46 (429.81)	1501.35 (472.91)	**<0.001**	1737.11 (365.86)	1803.01 (443.33)	0.060
Proteins (g/d)	58.25 (17.74)	60.12 (19.72)	0.178	56.49 (15.60)	72.29 (20.83)	**<0.001**
Carbohydrates (g/d)	179.45 (68.17)	200.80 (76.48)	**0.002**	165.45 (58.87)	246.67 (74.16)	**<0.001**
Free sugars (g/d)	84.65 (43.19)	34.48 (19.31)	**<0.001**	77.79 (40.34)	41.05 (25.53)	**<0.001**
Fats (g/d)	94.58 (13.26)	57.92 (22.23)	**<0.001**	93.87 (11.92)	67.44 (18.99)	**<0.001**
SFA (g/d)	24.46 (5.96)	20.77 (9.19)	**<0.001**	24.41 (5.40)	27.67 (9.05)	**0.003**
MUFA (g/d)	53.39 (4.82)	22.42 (9.85)	**<0.001**	53.12 (4.37)	25.03 (8.31)	**<0.001**
PUFA (g/d)	10.07 (1.48)	10.05 (6.73)	**<0.001**	9.85 (1.31)	9.36 (4.98)	**0.001**
Cholesterol (mg/d)	219.96 (78.20)	237.02 (139.57)	0.859	219.25 (68.16)	257.02 (135.31)	0.160
Fiber (g/d)	13.20 (4.73)	18.24 (7.77)	**<0.001**	11.94 (4.04)	21.55 (7.09)	**<0.001**
Calcium (mg/d)	683.13 (274.21)	565.31 (298.84)	**<0.001**	682.23 (252.82)	746.44 (332.10)	0.270
Iron (mg/d)	7.84 (2.74)	8.53 (2.79)	**0.001**	7.38 (2.28)	9.97 (2.98)	**<0.001**
Vitamin D (µg/d)	1.83 (1.12)	2.57 (3.67)	0.945	1.79 (1.02)	4.14 (7.71)	0.224
Vitamin E (mg/d)	10.13 (1.22)	7.92 (4.76)	**0.001**	9.92 (1.06)	7.54 (3.43)	**<0.001**
Vitamin C (mg/d)	80.46 (37.24)	67.81 (56.25)	**<0.001**	74.00 (34.89)	80.84 (66.91)	0.555
Vitamin B1 (mg/d)	0.95 (0.31)	0.89 (0.43)	**0.011**	0.90 (0.26)	1.07 (0.42)	**<0.001**
Vitamin B2 (mg/d)	1.36 (0.47)	1.27 (0.48)	**0.047**	1.34 (0.41)	1.48 (0.48)	0.061
Vitamin B3 (mg/d)	13.23 (4.56)	12.24 (5.66)	**0.049**	12.54 (3.87)	13.60 (5.61)	0.261
Vitamin B6 (mg/d)	1.35 (0.48)	1.78 (0.75)	**<0.001**	1.26 (0.38)	2.12 (0.86)	**<0.001**
Vitamin B9 (µg/d)	214.35 (77.87)	181.95 (86.60)	**<0.001**	198.44 (64.06)	211.34 (103.20)	0.891
Vitamin B12 (µg/d)	4.43 (1.61)	2.82 (1.95)	**<0.001**	4.44 (1.53)	3.97 (3.57)	**<0.001**

SFA, saturated fatty acids; MUFA, monounsaturated fatty acids; PUFA, polyunsaturated fatty acids. Statistically significant differences are highlighted in bold.

**Table 3:** Adequacy to the dietary recommendations of pregnant women from Spain and Poland.

	1st Trimester				3rd Trimester			
	RDA	Spain (n = 452)	Poland (n = 130)	*p*	RDA	Spain (n = 414)	Poland (n = 51)	*p*
Energy (Kcal/d)	2305	78.37 (18.65)	65.13 (20.52)	**<0.001**	2675	64.94 (13.68)	67.40 (16.57)	0.237
Proteins (g/d)	71	82.04 (24.99)	84.68 (27.78)	0.301	71	79.56 (21.97)	101.82 (29.34)	**<0.001**
Carbohydrates (g/d)	175	102.55 (38.95)	114.74 (43.70)	**0.005**	175	94.54 (33.64)	140.95 (42.38)	**<0.001**
Fiber (g/d)	28	47.14 (16.91)	65.15 (27.74)	**<0.001**	28	42.65 (14.45)	76.98 (25.33)	**<0.001**
Calcium (mg/d)	1000	68.31 (27.42)	56.53 (29.88)	**<0.001**	1000	68.22 (25.28)	76.64 (33.21)	<0.188
Iron (mg/d)	27	29.04 (10.17)	31.58 (10.33)	**0.013**	27	27.34 (8.45)	36.94 (11.01)	**<0.001**
Vitamin D (µg/d)	15	12.18 (7.46)	17.11 (24.46)	**0.025**	15	11.92 (6.77)	27.63 (51.40)	**0.034**
Vitamin E (mg/d)	15	67.53 (8.12)	52.77 (31.73)	**<0.001**	15	66.16 (7.04)	50.28 (22.84)	**<0.001**
Vitamin C (mg/d)	85	94.66 (43.81)	79.78 (66.19)	**0.008**	85	87.06 (41.04)	95.10 (78.72)	0.476
Vitamin B1 (mg/d)	1.4	67.87 (22.28)	63.64 (30.73)	0.145	1.4	64.62 (18.86)	76.66 (29.70)	**0.007**
Vitamin B2 (mg/d)	1.4	96.90 (33.92)	90.37 (34.20)	0.054	1.4	95.82 (29.40)	105.54 (34.42)	**0.030**
Vitamin B3 (mg/d)	18	73.51 (25.34)	67.98 (31.44)	0.067	18	69.69 (21.52)	75.53 (31.16)	0.199
Vitamin B6 (mg/d)	1.9	71.01 (25.06)	93.48 (39.61)	**<0.001**	1.9	66.52 (20.04)	111.71 (45.27)	**<0.001**
Vitamin B9 (µg/d)	600	35.72 (12.98)	30.33 (14.43)	**<0.001**	600	33.07 (10.68)	35.22 (15.20)	0.387
Vitamin B12 (µg/d)	2.6	170.23 (62.00)	108.57 (75.19)	**<0.001**	2.6	170.60 (58.85)	152.84 (137.47)	0.366

Data expressed as mean % (SD). RDA, recommended dietary allowance. Statistically significant differences are highlighted in bold.

**Table 4:** Adequacy of energy from different macronutrients in pregnant women from Spain and Poland.

	1st Trimester			3rd Trimester		
	Spain	Poland	*p*	Spain	Poland	*p*
Proteins (10–35%)	92.90	99.20	**0.006**	94.20	100.00	0.076
Below recommendation	7.10	0.80		5.80	0.00	
Above recommendation	0.00	0.00		0.00	0.00	
Carbohydrates (45–65%)	18.40	67.70	**<0.001**	11.40	82.40	**<0.001**
Below recommendation	81.60	21.50		88.60	9.80	
Above recommendation	0.00	10.80		0.00	7.80	
Total fats (20–35%)	0.20	54.60	**<0.001**	0.50	58.80	**<0.001**
Below recommendation	0.00	1.50		0.00	0.00	
Above recommendation	99.80	43.80		99.50	41.20	
SFA (<10%)	8.00	28.50	**<0.001**	4.60	13.70	**0.008**
Above recommendation	92.00	71.50		95.40	86.30	
MUFA (10–25%)	29.00	71.50	**<0.001**	20.40	78.40	**<0.001**
Below recommendation	0.00	24.60		0.00	21.60	
Above recommendation	71.00	3.80		79.60	0.00	
PUFA (5–10%)	56.00	36.90	**<0.001**	64.00	31.40	**<0.001**
Below recommendation	44.00	50.80		36.00	64.70	
Above recommendation	0.00	12.30		0.00	3.90	

SFA, saturated fatty acids; MUFA, monounsaturated fatty acids; PUFA, polyunsaturated fatty acids. Statistically significant differences are highlighted in bold.

## Data Availability

The data presented in this study are available on request from the corresponding author.

## Supplementary Materials

The following supporting information can be downloaded at: https://www.mdpi.com/article/10.3390/nu15143225/s1, Supplementary File S1. Food frequency questionnaire used in ECLIPSES study.
