# Supplementary material for: Nutrient Intake among Pregnant Women in Spain and Poland: A Comparative Analysis
Source: Nutrients. 2023 Jul 20;15(14):3225. doi: 10.3390/nu15143225 (PMC10386542; doi:10.3390/nu15143225)
Supplement: Supplementary file 1 [file nutrients-15-03225-s001.zip › nutrients-2501009-supplementary.pdf]

Supplementary File S1. Food frequency questionnaire used in ECLIPSES study.

Estudi ECLIPSES

ID: ☐☐ ☐☐ ☐☐☐ ☐

NOMBRE DE LA MADRE: ____________

NOMBRE DEL NIÑO/A: ____________

FECHA DE VISITA: ____________

ESTUDI ESCLIPSES-NEN: CUESTIONARIO DE ALIMENTACIÓN

HÁBITOS ALIMENTARIOS:

DE LA MADRE: INDIQUE CON UNA X LA RESPUESTA QUE USTED QUIERA SEÑALAR

1. ¿Qué tipo de leche toma habitualmente?:	1. Entera	2. Semidesnatada	3. Desnatada
2. ¿Qué tipo de yogur toma habitualmente?	1. Entera	2. Desnatada	3. Ambos
3. ¿Endulza con azúcar alimentos como leche, yogur,…?	1. No	2. Sí nº de cucharas_________	
4. ¿Qué tipo de pan toma habitualmente?	1. Blanco	2. Integral	3. Ambos
5. ¿Qué aceite utiliza habitualmente para aliñar?	1. Oliva	2. Semilla (girasol, maíz,..)	3.Indistintamente
6. ¿Qué aceite utiliza habitualmente para cocinar?	1. Oliva	2. Semilla (girasol, maíz,..)	3.Indistintamente
7. ¿Qué cantidad de aceite utiliza para cocinar?	1. Abundante	2. Moderada	3. Poca
8. En la mesa, ¿añade aceite a los platos (ejemplo: ensaladas,…)?	1. Habitualmente	2.Alguna vez	3. Casi nunca
9. ¿Le añade aceite en el pan o en los bocadillos?	1. Habitualmente	2. Alguna vez	3.Casi nunca
10. ¿Le añade mantequilla en el pan o en los bocadillos?	1. Habitualmente	2. Alguna vez	3.Casi nunca

DEL NIÑO: INDIQUE CON UNA X LA RESPUESTA QUE USTED QUIERA SEÑALAR

1. ¿Qué tipo de leche toma habitualmente?:	1. Entera	2. Semidesnatada	3. Desnatada
2. ¿Qué tipo de yogur toma habitualmente?	1. Entera	2. Desnatada	3. Ambos
3. ¿Endulza con azúcar alimentos como leche, yogur,…?	1. No	2. Sí nº de cucharas_________	
4. ¿Qué tipo de pan toma habitualmente?	1. Blanco	2. Integral	3. Ambos
5. ¿Qué aceite utiliza habitualmente para aliñar?	1. Oliva	2. Semilla (girasol, maíz,..)	3.Indistintamente
6. ¿Qué aceite utiliza habitualmente para cocinar?	1. Oliva	2. Semilla (girasol, maíz,..)	3.Indistintamente
7. ¿Qué cantidad de aceite utiliza para cocinar?	1. Abundante	2. Moderada	3. Poca
8. En la mesa, ¿añade aceite a los platos (ejemplo: ensaladas,…)?	1. Habitualmente	2.Alguna vez	3. Casi nunca
9. ¿Le añade aceite en el pan o en los bocadillos?	1. Habitualmente	2. Alguna vez	3.Casi nunca
10. ¿Le añade mantequilla en el pan o en los bocadillos?	1. Habitualmente	2. Alguna vez	3.Casi nunca

Estudi **ECLIPSES**

ID: ☐☐ ☐☐ ☐☐☐ ☐

NOMBRE DE LA MADRE: ______________________

NOMBRE DEL NIÑO/A: ______________________

FECHA DE VISITA: ______________________

Cuestionario de Frecuencia de consumo Alimentario – CFCA:

Este cuestionario le pregunta la **frecuencia con la que la madre y su hijo/hija consume de forma habitual** determinados alimentos. La frecuencia de consumo se debe especificar en los recuadros de la derecha del listado alimentos de este cuestionario.

Para cada alimento del listado debe apuntar el **número de veces** que lo consume.

•Si lo consume todos los días de la semana (una vez al día), ponga un 7 en la columna A LA SEMANA.

•Si lo consume alguna vez a la semana, ponga las veces: 1-2-3-4-5 o 6 en la columna A LA SEMANA.

Piense siempre en sumar el consumo de todas las comidas del día (desayuno, comida, merienda, cena , otros ..).

Por ejemplo:

- Si toma todos los días leche para el desayuno y alguna vez a la semana para cenar: 7 + 3 = 10 veces a la semana
- Si toma carne 4 veces a la semana para comer y 2 para cenar: 4 +2 =6 veces a la semana

Únicamente marque una casilla por cada alimento (A LA SEMANA o AL MES)

• Si consume el alimento alguna vez al mes, ponga las veces: 1-2-3 etc...en la columna: AL MES

• Si no lo consume nunca o casi nunca, deje la casilla en blanco, sin poner nada.

Estudi **ECLIPSES**

ID: [ ][ ] [ ][ ] [ ][ ][ ] [ ]

NOMBRE DE LA MADRE: ____________________

NOMBRE DEL NIÑO/A: ____________________

FECHA DE VISITA: ____________________

Cuestionario de Frecuencia de consumo Alimentario – CFCA
DE LA MADRE

¿CUANTAS VECES COME?	A LA SEMANA	AL MES
Leche		
Yogurt		
Chocolate: tableta, bombones, Kit-Kat, Mars…		
Cereales inflados de desayuno ("Corn-Flakes" "Kellog's")		
Galletas tipo "maría"		
Galletas con chocolate, crema…		
Madalenas , bizcocho		
Ensaimada, Donut, Croissant		
Ensalada: Lechuga ,tomate, escarola….		
Judías verdes, acelgas, espinacas		
Verduras de guarnición: berenjena, calabacín, champiñón.		
Patatas al horno, fritas o hervidas		
Legumbres: lentejas, garbanzos, alubias..		
Arroz blanco, paella		
Pasta: fideos, macarrones, espaguetis…		
Sopas y cremas		
Huevos		
Pollo o pavo		
Ternera, cerdo, cordero (bistec, empanada)		
Carne picada: longaniza, hamburguesa		
Pescado blanco: merluza, mero..		
Pescado azul: sardinas, atún, salmón….		
Marisco: mejillones, gambas, langostinos, pulpo, calamares…		
Croquetas, empanadillas, pizza		
Pan (bocadillos, en comidas)		
Jamón salado, dulce ,embutidos		
Queso blanco o fresco (Burgos,…) o bajo en calorías		
Otros quesos curados o semicurados, cremosos		
Frutas cítricas: Naranja, mandarina		
Otras frutas: Manzana, pera ,melocotón, albaricoque, plátano		
Frutas en conserva(en almíbar)		
Zumos de fruta natural		
Zumos de fruta comercial		
Frutos secos: cacahuetes, avellanas, almendras		
Postres lácteos: natillas, flan, cuajada		
Pasteles de crema y chocolate		
Bolsas de aperitivos ("chips", "chetos","fritos"…)		
Golosinas: caramelos, gominolas…		
Helados : en verano/en invierno		
Bebidas azucaradas ("coca-cola", "Fanta"…)		
Bebidas bajas en calorías (Coca-Cola light, cero…)		
Vino, sangría		
Cerveza		
Cerveza sin alcohol		
Bebidas destinadas (whisky, ginebra, coñac,..)		

Estudi ECLIPSES

ID: ☐☐ ☐☐ ☐☐☐ ☐

NOMBRE DE LA MADRE: ______________________

NOMBRE DEL NIÑO/A: ______________________

FECHA DE VISITA: ______________________

Cuestionario de Frecuencia de consumo Alimentario – CFCA
DEL NIÑO

¿CUANTAS VECES COME?	A LA SEMANA	AL MES
Leche		
Yogurt		
Chocolate: tableta, bombones, Kit-Kat, Mars...		
Cereales inflados de desayuno ("Corn-Flakes" "Kellog's")		
Galletas tipo "maría"		
Galletas con chocolate, crema...		
Madalenas , bizcocho		
Ensaimada, Donut, Croissant		
Ensalada: Lechuga ,tomate, escarola....		
Judías verdes, acelgas, espinacas		
Verduras de guarnición: berenjena, calabacín, champiñón.		
Patatas al horno, fritas o hervidas		
Legumbres: lentejas, garbanzos, alubias..		
Arroz blanco, paella		
Pasta: fideos, macarrones, espaguetis...		
Sopas y cremas		
Huevos		
Pollo o pavo		
Ternera, cerdo, cordero (bistec, empanada)		
Carne picada: longaniza, hamburguesa		
Pescado blanco: merluza, mero..		
Pescado azul: sardinas, atún, salmón....		
Marisco: mejillones, gambas, langostinos, pulpo, calamares...		
Croquetas, empanadillas, pizza		
Pan (bocadillos, en comidas)		
Jamón salado, dulce ,embutidos		
Queso blanco o fresco (Burgos,...) o bajo en calorías		
Otros quesos curados o semicurados, cremosos		
Frutas cítricas: Naranja, mandarina		
Otras frutas: Manzana, pera ,melocotón, albaricoque, plátano		
Frutas en conserva(en almíbar)		
Zumos de fruta natural		
Zumos de fruta comercial		
Frutos secos: cacahuetes, avellanas, almendras		
Postres lácteos: natillas, flan, cuajada		
Pasteles de crema y chocolate		
Bolsas de aperitivos ("chips", "chetos","fritos"...)		
Golosinas: caramelos, gominolas...		
Helados : en verano/en invierno		
Bebidas azucaradas ("coca-cola", "Fanta"...)		
Bebidas bajas en calorías (Coca-Cola light, cero...)		
